# Forgo or Go for One? The Unavailable Effect in Non-comparable Choice Sets

**DOI:** 10.3389/fpsyg.2019.01257

**Published:** 2019-06-06

**Authors:** Jing Tian, Rong Chen, Feng He

**Affiliations:** ^1^ Department of Marketing,Tsinghua University, Beijing, China; ^2^ Department of Economics and Trade, University of Science and Technology Beijing, Beijing, China

**Keywords:** unavailable effect, non-comparable choice set, which-to-buy mindset, whether-to-buy mindset, construal level

## Abstract

This research aimed to explore how consumers’ purchase behavior varies when they are faced with unavailable options in a non-comparable choice set. We investigated the unavailable effect based on goal-related mindsets theory and found that consumers with an activated *which-to-buy* mindset show higher purchase intention for the remaining options relative to those who have a *whether-to-buy* mindset. Four between-subject experiments were undertaken. Study 1 (including two experiments, both two groups) depicted the relationship between the mindset and consumer purchase choice. Study 2 examined the construal level as the underlying mechanism. Two further studies enabled two methods, such as shopping cart state and payment type, to activate different mindsets and found the boundary conditions of each method. Study 3 found that empty cart (vs. non-empty cart) activate whether-to-buy mindset restraining purchase intention, while the habitual (vs. non-habitual) initial purchase moderated the shopping cart effect. Study 4 found that paying by gift cards (vs. gifted cash) primed which-to-buy mindset increasing purchase intention, while the payment effect declined when the product was high in feasibility (vs. desirability). The insights gained from this research can guide both online and offline retailers in how to strategically manage consumer mindsets under unavailable circumstances. Optimal presenting timing and method of unavailable information may activate a different mindset and help boost sales of the remaining options at the same time.

## Introduction

When making purchase decisions, consumers often face situations where certain options are sold out but are still presented to them, for example in online purchasing, especially when large discounts are on offer, as stocks disappear sellers mark options as unavailable using an “out-of-stock” stamp. Although consumers are not able to choose the sold-out options, displaying the sold-out information changes the decision environment and has a great impact on consumer behavior. The *unavailable option* effect on consumer decision-making has aroused some research interest over the years (Kivetz and Simonson, 2000; Choplin and Hummel, 2005; Pettibone and Wedell, 2007; Chuang et al., 2012). For example, some studies show that adding an option that asymmetrically dominates a targeted alternative, while actually being unavailable, increases preference for the target in the original choice set (Choplin and Hummel, 2005; Pettibone and Wedell, 2007). However, the contexts above are all focused on the comparable attributes of products, such as quality and price, which can be ranked with certain rules (Nowlis and Simonson, 1997). But what if the options are equal in comparable attributes, while differ in some non-comparable features, such as design or taste? For example, the availability of several color options for a series of coats, which are identical in price and quality, or several flavors of chewing gum from the same brand at the same price and volume. Little is known about how an unavailable option influences decisions in a common non-comparable choice set. How would consumers evaluate the remaining options and make a decision? Under what circumstances consumers will forgo or go for one option, and what should retailers do to boost consumers’ choice of remaining options? This phenomenon is most common in the daily consumption sector, and the effects are of most concern to individual companies. Realizing both the theoretical gap and practical relevance, we aim to examine this issue.

We examine the questions based on goal-related mindsets theory and posit that the type of mindset determines a goal-related purchase decision. In particular, consumers with an activated *which-to-buy* mindset focus on the differences between the options, which are difficultly compared and ranked in non-comparable attributes, thus increasing their purchase intention for the remaining options. Correspondingly, consumers with a *whether-to-buy* mindset would pay attention to the incomplete structure of the choice set, thus increasing forgo intention. Construal level is proved to be the underlying mechanism in the relationship between mindset and purchase intention. Guided by this basic hypothesis of goal-related mindset, we further find two indicators that should activate the different mindsets. Firstly, since an initial purchase will activate consumers’ mindsets from deliberative to implementation (Dhar et al., 2007), we expect shopping cart state to represent different purchase stages and to prime different types of mindsets. Consumers with an empty shopping cart will orientate a whether-to-buy mindset and show lower purchase intentions compared with those who have bought something activating the which-to-buy mindset. The effect of mindset activated by the shopping cart is expected to be moderated by initial purchase type. Finally, we examine the payment type as another method activating different mindsets. Yao and Chen (2014) showed that gift card recipients appear to confront a concrete task compared with recipients of gifted cash. We posit that consumers paying by gift cards (vs. gifted cash) will show higher purchase intention for the remaining options since gift cards activate the which-to-buy mindset (vs. whether-to-buy mindset). The effect of mindset activated by payment type should be declined when the product performs high in feasibility comparing with desirability.

Across a series of experiments, we showed the relationship between mindset and purchase intention of remained options in the non-comparable choice set with unavailable options. In particular, we found that consumer purchase choice may decline when they are activated whether-to-buy mindset, compared with when activated with which-to-buy mindset (Experiments 1a and 1b). We further demonstrated that the underlying mechanism of construal level can explain the effect of mindset on purchase intention of remained options (Experiment 2). Additionally, we found two context variables that prime different mindsets and subsequently affect purchase intention (Experiments 3 and 4). In Experiment 3, we manipulated the shopping cart state to prime different mindsets and found the initial purchase as a moderator. Consumers with something in the cart in which-to-buy mindset are likely to go for the remained options, while those with empty carts in whether-to-buy mindset are likely to forgo or postpone purchasing. Habitual initial purchase (vs. non-habitual) decreased the effect of the shopping cart. In Experiment 4, we manipulated the payment type to prime different mindsets and examined the product type as a moderator. Consumers paying by gift card activated which-to-buy mindset are likely to take the remained options, comparing with those paying by gifted cash activated whether-to-buy mindset. The interaction effect was that products with high feasibility undermined the effect of payment type. The insights gained from this research can guide both online and offline retailers on how to strategically manage consumer mindsets under unavailable circumstances. For example, retailers can optimize the presenting method and timing like this, offline shopping guides could ask questions about preference, which can activate the which-to-buy mindset of consumers, while in the online context, the unavailable information ought to be presented after consumers choose the option, so that they stay in a which-to-buy mindset, thus increasing their intention to go for one. Besides, the payment type and the product desirability might be considered in order to boost sales.

The remainder of this paper is organized as follows. We begin by reviewing relevant literature on unavailable effect, goal-related mindsets, and construal level, from which we develop main hypotheses and main model. Then, we propose two variables to activate different mindsets and bring out corresponding boundary conditions, presented in moderating model. Four studies are conducted to examine the theoretical hypothesis and models. Experiments 1a, 1b, and 2 establish the main effect and examine the underlying mechanism. Experiments 3 and 4 support the moderating model. We conclude by discussing the theoretical and managerial implication, as well as some possible directions for future research.

## Theoretical Background

The notion of unavailable effect in the decision context has received considerable attention. Most previous studies can be broadly classified into two streams. One investigates the effect when the target product is unavailable; finding that an unavailability results in delaying, switching, or even forgoing (Tversky and Shafir, 1992; Greenleaf and Lehmann, 1995; Corsten and Gruen, 2003; Gunasti and Ross, 2008). Furthermore, Dhar and Simonson (2003) have explored which products lose market share easily when unavailable. The other stream focuses on the effect on the target product when other products are unavailable. Parker and Schrift (2011) demonstrated that an unavailable option in a choice set will change consumers’ attention and evaluation criteria and increase the importance of dominant attributes. Boland et al. (2012) have shown that if the top choice is unavailable, a consumer does not necessarily move to the second-ranked choice, since the preference from the initial “screening” attributes might change after comparison based on “differentiating” attributes. Both streams of research have achieved supportive findings. However, the above studies all focused on the comparable attributes of products (e.g., price, quality; Markman and Medin, 1995), which produce precise and easy-to-compute comparisons, and can be ranked with certain rules (Nowlis and Simonson, 1997). Cho et al. (2013) found that decision difficulty is associated with mental representation, which differs for comparable and non-comparable choice sets. In this research, we focus on the consumer decision when there are some unavailable options in a non-comparable choice set.

### The Influence of Mindset

The impact of mindset begun to attract attention in consumer research in recent years, which has been stimulated by the research of decision-making conducted by Gollwitzer and his collaborators. They proposed that goal-oriented behavior can be separated into two stages: in the pre-decision stage, individuals generate pros and cons and develop a deliberative mindset; once a choice between goals is made, individuals enter the post-decision stage, in which they adopt an implemental mindset that considers where, when, and how to act (Gollwitzer et al., 1990). Once activated, these mindsets persist to impact reactions to subsequent behavior. Briley and Wyer (2002) proved that individuals activated as a group member behaved in a prevention focus mindset, and they preferred to avoid potentially negative consequences of decisions; for example, they were likely to choose more kinds of candies in case of making the wrong choice. Lee and Ariely (2006) demonstrated that consumers spontaneously acquire different mindsets at different stages of a shopping experience and found that promotions have a greater effect when consumers are at the initial stage than when they are clear about their purchase decisions. Moreover, Xu and Wyer (2007) proposed the theory of goal-related mindsets that consumers have a general shopping procedure or script stored in memory, which consists of a series of sub-goals of deciding which to buy and deciding whether to buy. The sub-goals are normally activated and applied in sequence, and the achievement of one goal is more likely to stimulate the goal following it rather than the goal preceding it. Chandran and Morwitz (2005) showed that inducing an implemental mindset can increase participants’ likelihood of purchasing. Most of them discussed the influence of mindset on target products choice, while limited research focused on the choice of remaining options when some other options are unavailable.

We infer that when consumers face the unavailable option in a non-comparable choice set, if the which-to-buy mindset is activated, they would focus on comparing the features of different options. Since the features are non-comparable, we expect the likelihood of purchasing the remaining options to be increased. If they are primed with a whether-to-buy mindset, they would pay attention to the incomplete structure of the choice set. While being forced to choose induces higher depletion than making an autonomous choice (Moller et al., 2006), the higher self-depletion may strengthen their intention to forgo by postponing the purchase or switching to another choice set.

**H1**: When faced with an unavailable option in a non-comparable choice set, consumers who have an activated which-to-buy mindset show higher intention to buy a remaining option compared with those who have a whether-to-buy mindset activated.

### Underlying Mechanism of Construal Level

It has been theoretically well established that cognitive procedure is represented in memory as a sequence of temporally related segments (Srull and Wyer, 1989), which are called upon to guide goal-directed behavior. In general shopping, consumers maintain a script in their memory, which consists of a series of sub-goals for deciding which to buy and deciding whether to buy (Xu and Wyer, 2007). The first sub-goal, at the pre-decision stage, allows for more abstract, global processing, and high-level construal, whereas the second sub-goal, at the post-decision stage, allows for more detailed, local processing and low-level construal (Förster and Dannenberg, 2010). Thus, consumers with an activated whether-to-buy mindset are likely to construe unavailable information at a higher level, paying more attention to the whole structure of the choice set. In contrast, a which-to-buy mindset may induce a lower construal level, keeping consumers’ eyes on the specific residual options, which perform equally in the functional and comparable attributes.

**H2**: Construal level mediates the influence of different mindsets on purchasing decisions. Consumers who have an activated whether-to-buy mindset construe information at a higher level than those who are activated with a which-to-buy mindset.

**Figure 1:**
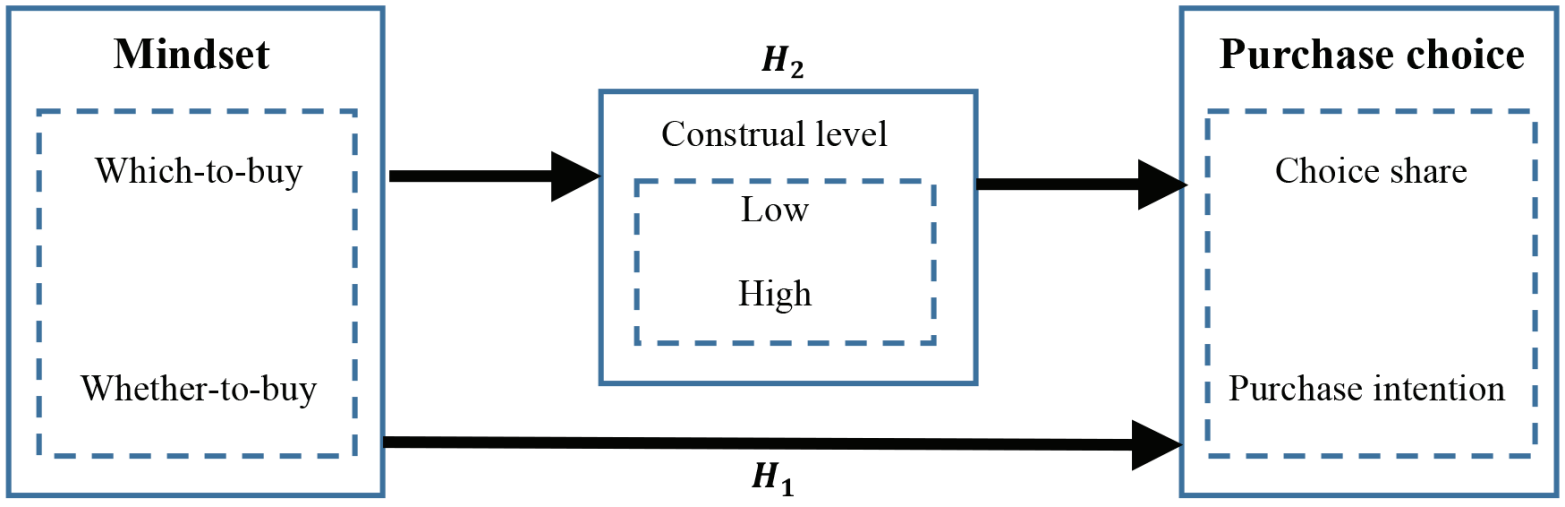
Main model.

The main theoretical model is presented in Figure 1.

## Activating Mindsets and Boundaries

**Figure 2:**
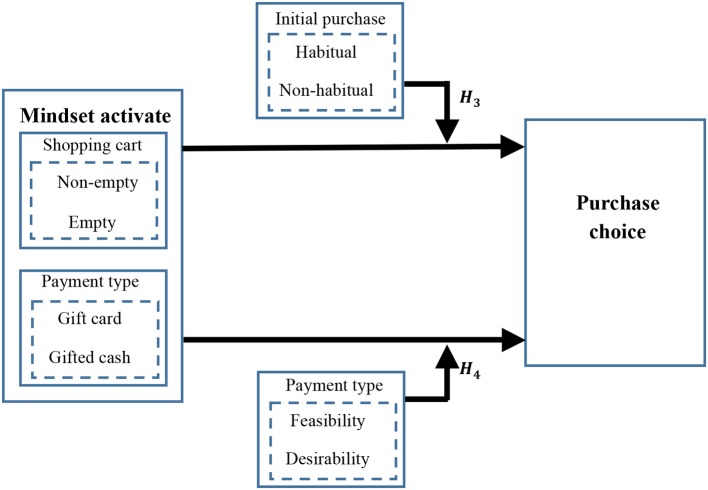
Moderating model.

After proposing the relationship and mechanism between mindset and purchase intention in the above, to find some managerial variables which can be manipulated is essential. These enable retailers to pursue specific strategies to promote sales of remaining options with stock out options. Some context variables have been shown to influence consumers’ subsequent goal and information processing. Once certain mindsets activated, these mindsets persist to influence reactions to subsequent activities (Gollwitzer et al., 1990). What is more, mindsets can be induced either directly, by performing a task that requires thinking about how to attain a goal, or indirectly, by engaging in an unrelated participative exercise (Taylor and Gollwitzer, 1995). We propose two context variables: shopping cart state (empty vs. non-empty) and payment type (gifted cash vs. gift card) that would activate whether-to-buy/which-to-buy mindsets. Moreover, we expect to find the boundaries of a different effect, in order to help retailers to adopt promotion procedures in the target to a different context. The moderating model is presented in Figure 2.

### Shopping Cart States Activate Mindsets

Dhar et al. (2007) found that making an initial purchase increased the likelihood of making a later purchase in an unrelated domain. The initial purchase would push consumers’ mindsets from deliberative to implementation, evoking feelings of commitment to purchase by reducing the psychological barriers to action, thus increasing the intention for a later purchase. Based on this momentum effect, we expect that while the shopping cart is empty, consumers remain in the first stage to consider whether to buy certain items and that unavailable information may restrain their subsequent behavior. Once they have added some goods into the cart, the implementation mindset is continued, and they tend to overlook the unavailable option and think about which item to choose from the remaining options.

If an initial purchase in a shopping cart does indeed increase the purchase intention for remaining options, will the effect be greater for certain types of initial purchase than others? Here, we consider the distinction between habitual and non-habitual purchases. Assael (1981) classified four types of consumer purchase: complex, choice, loyal, and habitual, based on two dimensions that are purchase involvement and the degree of difference between competing brands. Of these purchase types, the habitual purchase decisions have the lowest involvement and difference degree and consumers can purchase without much deliberation. As Jacoby and Kyner (1973) pointed out, the habitual purchase was not necessarily the result of a strong positive brand evaluation. Rather, it represents a convenient way of reducing cognitive effort. We expect that consumers making a habitual initial purchase to maintain a whether-to-buy mindset, and thus, their subsequent purchase intention for the remaining options in a non-comparable set will not increase. That is, when the initial purchase is habitual, the increasing effect of making an initial purchase will decline.

**H3**: The effect of mindset activated by shopping cart state is greater when the initial purchase is not habitual. When the initial purchase is habitual, the effect will be undermined.

### Payment Types Activate Mindsets

In addition to previous shopping behavior, payment is an important factor for consumers making a purchase decision. Mental accounting theory posits that individuals are accustomed to categorizing money into accounts based on a hierarchy of types of expenditure (Shefrin and Thaler, 1988). White (2008) suggests that gifted cash is allocated with other current assets, whereas gift cards are perceived as more “spendable” and not viewed as “real” money, since gift cards only realize utility when redeemed. In addition, gift card recipients appear to confront a concrete task – that is which to buy, and when, where, and how to redeem the card – compared with the gifted cash recipients (Yao and Chen, 2014). Thus, consumers paying with gift cards are oriented to adopt a which-to-buy mindset, while those paying with gifted cash are free to decide whether to buy. We can infer that consumers paying in gifted cash have lower purchase intentions for remaining options than those paying with a gift card.

Building on the hypothesis above, we consider whether the payment effect will be greater for certain types of purchase than others depending on the feasibility and desirability of the product. The distinction between desirability and feasibility corresponds to the distinction between means and ends (Gollwitzer and Moskowitz, 1996; Kruglanski, 1996). Desirability refers to the valence of an action’s end state, whereas feasibility refers to the ease or difficulty of reaching the end state (Liberman and Trope, 1998). We expect that consumers faced with a product high in feasibility tend to construe at a lower level. When the feasibility of product is high, even paying in gift cash, consumers focus on the non-comparable attributes of options, and their purchase intention for the remaining options will not decrease much. That is, the payment type effect will be reduced for high feasibility products.

**H4**: The effect of mindset activated by payment type is greater for desirable purchases than for feasible purchases. When the product is high in feasibility, the effect will be undermined.

## Research Design

In view of difference in consumer preference for different options of a product, if consumers show a strong preference for an unavailable option, the unavailable option will directly have a strong influence on purchase intention, so the research plans to exclude options showing obvious difference in preference to ensure that consumers show no obvious preference for options of the product. As mentioned in the research by Fay and Xie (2008) on probabilistic goods, consumers show different preference intensity and probabilistic goods sell to consumers with uncertain preference to relieve the contradiction between supply and demand. The research focuses on scenarios without obvious preference and controls the degree of preference for different options through a preliminary experiment.

The preliminary research requested respondents to give each of the 12 colors of the same headphones a preference score. In order to avoid a unanimous high preference for an option, the research asked respondents to list three favorite options after scoring on the five-point scale. At the end of the questionnaire, respondents were inquired about gender. The preliminary research was carried out by Qualtrics and collected 34 effective questionnaires, with male respondents accounting for 58%. After taking into comprehensive consideration the mean and variance of preference scores and mentions of “favorite options” (*T*), six colors are screened out (*M* > 2.5; SD < 1.20; *T* > 5), effectively meeting the precondition of no obvious preference for the six options when subjects are choosing products in subsequent main research.

Based on preliminary research, we would carry out four studies subsequently to test hypotheses regarding the effects of “non-comparable unavailability” on the purchase intention. The questionnaire would measure the purchase intention based on a scale revised by Putrevu and Lord (1994) and contained items concerning the measurement of purchases. In order to better measure the effects, as previous research found that presenting sold-out options would increase the choice share of the available options (Kramer and Carroll, 2009), we also used choice share to measure the purchase behavior.

## Study 1

The purpose of study 1 was to test the influence of mindset on purchase intention of remaining options. We manipulated the participants’ mindset by asking them to state preferences for one type of product (without making purchasing decisions) or to decide whether to make a purchase at the set of products (without stating preferences). Experiments 1a and 1b were conducted in different samples and measured in a different way to support the main hypothesis.

### Experiment 1a

#### Participants and Design

Sixty students (31 males, 29 females, *M*_age_ = 21.75 years) from a university enrolled in this study, which adopted a between-subjects (which-to-buy mindset vs. whether-to-buy mindset) design. Participants were randomly assigned to one of the two conditions, and each was given a gift valued at 5yuan. Gender and age did not interact with the independent variable in this and subsequent studies and were therefore excluded from consideration.

#### Procedure

Participants received information about two types of products, each described by six features: three positive and three negative. In one condition, participants first decided whether they would want to choose one of the two types of products or would rather not chose any kind at all, thus activating a whether-to-buy mindset. Then, after making a decision, they had to consider the second decision in a non-comparable choice set. In the other condition, participants first indicated which of the two types of products they preferred, thus activating a which-to-buy mindset.

Firstly, participants read the following scenario:

Recently, I feel that my old headphones cannot meet my needs. I typed in ‘headphone’ online, trying to find a new set to buy. Below the search bar, numerous headphones are displayed.

Then, a picture of eight headphones is shown, four of which are earphones, while the other four are headsets. The advantages and disadvantages of the two kinds of headphones are described in the following instructions:

“The advantages of the headset are as follows: the sound field is good; comfortable to wear; out of the ear avoids damaging ear canals; strong and lasts longer. Headsets also have drawbacks: out of the ear, the bass is not very good; inconvenient to carry; need to be driven by powerful equipment, like computers.”

In the whether-to-buy condition, participants were asked to indicate “whether they would choose one kind of headphones or not.” While in the which-to-buy condition, they were asked to indicate “which kind of the two they would prefer.” Then, all participants were shown the non-comparable choice set as follows:

“After comparing between many stores, I found these headphones, the quality and price of which fit my need. The instructions are as follows: This headphone is designed with a patent, adjustable, and comfortable to wear. The earmuffs are crafted from artificial leather, soft and well fitting round the ears, with good permeability. It uses a special long-handled microphone with high sensitivity and multi-wire design. It has superior resolving power, showing strong detail performance, full bass, calm midrange, and clear treble. It is not only a good aid for study and work but also can meet your needs in the world of music and games. This headset is designed in six colors, two of which have sold out, but the other four colors are still available.”

Besides the text description, a picture of the six colors of headphones was displayed, in which two were marked with “sold out” tags. Participants were asked about their purchase intention for the remaining options in the choice set, by responding to the question “I will purchase this headphone” and “I will purchase this headphone now” along with a 7-point scale (1 = not at all and 7 = definitely agree). After reporting their purchase intention, we also measured the cognitive dissonance, decision difficulty, and information complexity level through three items with 7-point scales (Freides, 1974; Sweeney et al., 2000; Goodman et al., 2013). Then, we collected the demographic information, thanked, and dismissed them.

#### Result

**Figure 3:**
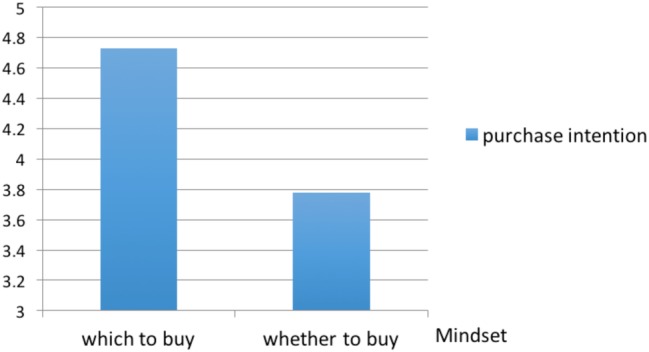
Purchase intention in different mindset condition.

We ran an ANOVA with the type of mindset activated as the independent variable and the purchase intention as the dependent variable. We created a purchase intention index by averaging the two intention items (*α* = 0.72). The results indicated a significant main effect of mindset type [*F*(1, 57) = 4.34, *p* = 0.04]. As predicted, participants who had a which-to-buy mindset activated showed higher intention to buy a remaining option (*M* = 4.73, SD = 1.79) than those who had a whether-to-buy mindset activated (*M* = 3.78, SD = 1.68) (see Figure 3). To rule out some confounding of alternative explanations, we have conducted a post-test check. And we found no significant difference in cognitive dissonance [*F*(1, 57) = 0.228, *p* = 0.635], decision difficulty [*F*(1, 57) = 0.305, *p* = 0.583], and information complexity level [*F*(1, 57) = 1.008, *p* = 0.319].

### Experiment 1b

#### Participant and Design

Ninety-five participants recruited from Amazon MTurk (49% female; *M*_age_ = 36.9, SD = 10.1) and participated in this experiment in exchange for a small monetary incentive. Participants were randomly assigned to one of the two conditions: which-to-buy mindset vs. whether-to-buy mindset. Gender and age did not interact with the independent variable in this and subsequent studies and were therefore excluded from consideration.

#### Procedures

Participants received information about two types of products, both are described with six features. In the which-to-buy condition, participants first decided whether they would want to choose one or rather not choose any kind at all, thus activating a whether-to-buy mindset. In the whether-to-buy condition, participants first indicated which of the two types of products they preferred, thus activating a which-to-buy mindset.

After this, they went ahead to consider another decision in a non-comparable choice set. The non-comparable choice set was shown through text description and picture as experiment 1a. Participants were then asked to indicate whether they would like to choose one of the available options or defer their choice to a later time. Finally, participants reported their gender and age.

#### Results

We tested whether the mindsets would influence the choice share of the consumers (i.e., not to defer their choice to a later time) using a crosstabs analysis. As predicted, 95.6% (43 out of 45) of those in the which-to-buy condition decided to purchase, which was significantly larger than that in the whether-to-buy condition as only 78% (39 out of 50) decided to purchase.

#### Discussion

The findings of experiment 1a and 1b were consistent with H1. Since the features of options within a non-comparable choice set are hard to rank, if participants focus on comparing the features of different options after having a which-to-buy mindset activated, the likelihood of purchasing the remaining options would increase. If they are primed with a whether-to-buy mindset, they would pay attention to the incomplete structure of the choice set, which may decrease their purchase choice (both purchase intention and choice share) for the remaining options. The next study will further explore the underlying mechanism.

### Experiment 2

Experiment 2 was conducted to test H2 and offer evidence for the underlying mechanism that different mindset types construe information at different levels. We achieved this goal through an incidental classification task after participants had indicated their purchase intention for remaining options. The classification task has been found to be effective at measuring construal level (e.g., Liberman et al., 2002; Hansen et al., 2012). Consumers who construe information at a high level will use fewer categories to classify objects in comparison to those who construe information at a low level.

#### Participants and Design

Sixty-two students (36 males, 26 Female; *M*_age_ = 21.6 years) from a university participated in the experiment. They were randomly assigned to one of the two conditions (which-to-buy mindset vs. whether-to-buy mindset).

#### Procedures

We used the same priming task as in Study 1 to activate which-to-buy and whether-to-buy mindsets. After participants indicating their purchase intention for the remaining headphone options, they were asked to complete a classification task for objects that they would take with them on a camping trip, as follows:

“Imagine that you are going with your family on a camping trip and you are thinking about what to bring. Take a look at the following items and place them into groups by writing down the items that belong together and then circling the items that belong in the same group. Please make sure to include every item, even if you would not use it in reality. Also, please do not overlap; that is, place each item in only one group. The items were fishing pole, cigarettes, bathing suit, brush, tent, matches, hat, soap, gloves, shovel, flashlight, sunglasses, marshmallows, snorkel, shirts, sweater, sneakers, coat, raft, boots, hot dogs, toothbrush, potato chips, blanket, dog, pants, socks, rifle, shoes, rope, sleeping bag, underwear, insect repellent, canteen, camera, beer, pillow, axe.”

After completing the task, the participants were asked about the purpose of the study but nobody correctly guessed.

#### Results and Discussion

Firstly, we ran an ANOVA with mindset type as the independent variable and purchase intention as the dependent variable. The results indicated a significant main effect of mindset type [*F*(1, 62) = 5.40, *p* < 0.05]. As predicted, participants with an activated which-to-buy mindset show a higher intention to buy a remaining option (*M* = 4.78, SD = 1.64) than those with an activated whether-to-buy mindset (*M* = 3.93, SD = 1.20). The finding replicated the results of Experiment 1.

Next, a series of analyses were conducted to examine the role of construal level as a potential mediator of the mindset effect. We coded the construal level index from the classification task using their group numbers; the more groups they classified, the higher the construal level index was, and the lower the construal level they adopted. To examine the underlying process, we tested H2 by using a procedure from Preacher and Hayes’s (2004) and Hayes (2013). The analysis with purchase intention as the dependent variable, construal level as the mediator, and the mindset as the independent variable revealed that (1) the indirect path (*B* = 0.2) with 95% confidence interval excluded 0 (0.03, 0.45). This result supported H2, suggesting that consumers’ construal level significantly mediated the relationship between mindset type and purchase intention for remaining options. Besides, when we controlled the construal level, the influence of mindset on purchase intention was not significant, with the path included 0 (−0.02, 0.74). It supports the hypothesis that consumers with an activated whether-to-buy mindset are likely to construe unavailable information at a high level, while the which-to-buy mindset may induce a lower construal level, and the construal level totally mediates the effect of mindset on purchase intention. The latter keeps consumers’ eyes on the specific residual options, which have equal performance in the functional and comparable attributes, thus increasing their purchase intention for the remaining options.

### Experiment 3

Experiment 3 was designed to test H3. We used the shopping cart state to activate different mindsets, which will influence the purchase intention for remaining options in a non-comparable choice set. We proposed that product type will be a moderator so that when the antecedent purchase is habitual, the carryover effect will be reduced.

#### Participants and Design

One hundred and fifty-five MBA students (97 males, 58 females, *M*_age_ = 25.6 years) participated in this study in exchange for credit. This study adopted a two [mindset type: empty (whether-to-buy) vs. non-empty (which-to-buy)] by two (initial purchase: habitual vs. non-habitual) between-subjects design. Two students did not complete the experiment and were excluded.

#### Procedures

We manipulated the initial purchase by presenting different products. In the habitual condition, participants were asked to imagine: “I like to buy toothpaste regularly and I want to stock up on tubes of toothpastes now. I type in “toothpaste” online. Below the search bar, numerous kinds of toothpaste are displayed.” In the non-habitual condition, participants were asked to imagine the following situation: “It is getting colder in Beijing, I want to buy some cotton socks to keep warm. I type in “socks” online. Below the search bar, numerous socks are displayed.” Since the experiment was conducted in late autumn, cotton socks were seasonal to students. After seeing the picture of toothpaste or socks, as well as the price and overview of each one, in the non-empty cart condition, participants were asked to indicate which one they preferred and would decide to buy. In the empty cart condition, participants were informed: “Since I can buy the toothpaste/socks conveniently in the supermarket in the university, where I can smell/touch them and get them immediately, I close the webpage of the toothpaste/socks and go on scanning other products.”

Having done so, all participants were shown the follow-up scenario, “My computer happens to play a beautiful song, which reminds me that I want to buy new headphones.” Then, the same unavailable scenario used in the previous studies, including the product information, was displayed to participants, and they were asked about their purchase intention toward the remaining options. Then, participants were required to rate a 2-item, 7-point scale anchored by mostly which to choose/whether to choose and mostley which to buy/whether to buy. Finally, demographic information was collected, and then we thanked and dismissed them.

#### Results and Discussion

Before the result analysis, we tested whether the manipulation was successful. As predicted, the results of a pretest indicated that the message with non-empty shopping cart state was deemed more which-to-buy mindset (*M* = 4.96, SD = 1.90) than that with the empty shopping cart [*M* = 4.27, SD = 1.32, *F*(1, 151) = 6.69, *p* = 0.01].

**Figure 4:**
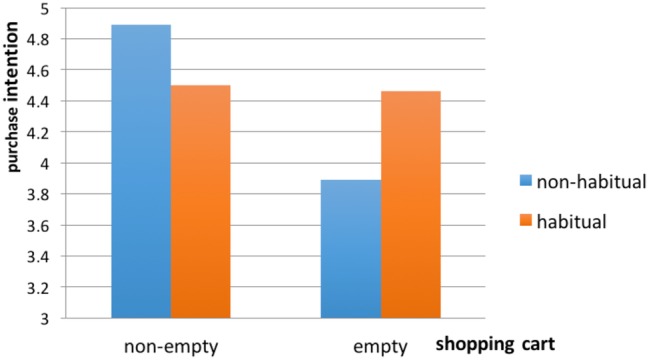
Interaction of habitual purchase and shopping cart.

Then, we conducted a GLM analysis with the type of mindset type (whether-to-buy: empty vs. which-to-buy: non-empty) and initial purchase (habitual vs. non-habitual) as the independent variables and purchase intention (*α* = 0.76) as the dependent variable. The main effect of mindset activated by the shopping cart state was marginally significant [*F*(1, 151) = 3.657, *p* = 0.058]. Participants with something in their shopping cart showed higher purchase intentions (*M* = 4.68, SD = 1.74) toward the remaining options than those with an empty shopping cart (*M* = 4.18, SD = 1.61). Consistent with our expectations (H3), the interaction between the mindset and the initial habitual purchase was marginally significant [*F*(1, 151) = 3.132, *p* = 0.079]. Participants in the non-habitual initial purchase condition showed higher purchase intentions when they bought something (*M* = 4.89, SD = 1.55) relative to those whose shopping carts were empty [*M* = 3.89, SD = 1.54, *F*(1, 72) = 7.687, *p* = 0.007]. In contrast, the effect of the shopping cart type was not significant for participants in the habitual initial purchase condition [*F*(1, 77) = 0.009, *p* = 0.923] (see Figure 4).

Since the shopping cart status represented buyers’ thinking stage, if they bought something, they went into the second stage – thinking about which to buy – and the likelihood of purchasing the remaining options would be increased. If the shopping cart was empty, they would stay in the first stage – considering whether to buy anything – and their purchase intention toward the remaining options was lower. The effect of mindset activated by shopping cart state was moderated by initial habitual purchase. When participants considered a habitual initial purchase, they did not need to make a complex two-stage decision. Even though they put something into shopping carts, the which-to-buy mindset was not successfully primed, and the effect of shopping cart state disappeared.

### Experiment 4

Experiment 4 was designed to test the effect of another context variable, *payment type*, on the purchase intention for the remaining options. We expected that participants paying with a gift card would activate a which-to-buy mindset and show higher intention to buy a remaining option compared with those using gifted cash. We examined product type as a moderator so that when the product is high in desirability (vs. feasibility), the effect of the payment type will be reduced (H4).

#### Participants and Design

One hundred and fifty-eight undergraduate students (63 males, 95 females, *M*_age_ = 20.4 years) participated in this study, which adopted a two [mindset type: gifted cash (whether-to-buy) vs. gift card (which-to-buy] by two (product: feasibility vs. desirability) between-subjects design. All students were randomly assigned to the four conditions and worked through the procedures on desk computers in the laboratory. Seven participants reported identical answers for all or most questions and were excluded.

#### Procedures

Participants first read the scenario:

“It was your birthday several days ago, and your closest aunt in Beijing gave you a 500 yuan gift card (cash) as a birthday gift. The gift card can be used at any store in one of the most famous online malls. Soon after your birthday, you placed the gift card (cash) in your wallet, intending to use it later on.” We also manipulated the payment type with different pictures, one was a picture of a gift card with a tag of 500 yuan, the other one was a picture of five 100 yuan bills. We guided the participants to imagine a shopping situation as follows: “Several days later, there is spare time for you to scan the online mall. You intend to make your purchase with the gift card (cash) from your aunt. Your computer happens to play a beautiful song, which reminds you that you want to buy new headphones.” Participants were shown the non-comparable choice set, “After comparison between many stores, I found the headphone, the quality, and price of which fit my need.”

We manipulated product type by describing the product using different information (Kim et al., 2009). In the high feasibility condition, we described the headphone as follows: “This headphone is normal style, adjustable, and comfortable to wear. It uses a long-handled microphone and multi-wire design. It can be folded and easily carried. Order now, we will dispatch it today and it can be delivered in 2–3 days.” In the high desirability condition, we described it like this: “This headphone is fashion style, with artificial leather earmuffs, soft and well-fitting round ears, with good permeability. It has superior resolving power, showing strong detail performance, full bass, calm midrange, and clear treble. You need to order it in advance. After dispatch, you will receive it within 5–7 days.” Below the information, six colors of the headphones were displayed, in which two were marked with “sold out” tags and the other four colors were still available. Participants were then asked about their purchase intention for the remaining options in the choice set. *Then participants were required to rate a 2-item, 7-point scale anchored by mostly which to choose/whether to choose and mostly which to buy/mostly whether to buy.* To check the manipulation on feasibility, the experimenter asked participants to rate their perceived feasibility on a seven-point scale: “To buy this headphone is a feasible decision” (1 = very disagree; 7 = very agree). Finally, demographic information was collected.

#### Results and Discussion

Before result analysis, we tested whether the mindset type was successfully manipulated. As predicted, the results of a pretest indicated that the message of using gift card was deemed more which-to-buy mindset (*M* = 4.85, SD = 1.44) than that with gifted cash [*M* = 4.15, SD = 1.59, *F*(1, 149) = 8.034, *p* = 0.005]. There was also a significant difference of the experimental manipulation on perceived feasibility [*F*(1, 149) = 10.296, *p* = 0.002]. The participants in the high feasibility condition perceived high feasibility (*M* = 5.21, SD = 1.35) compared with the participants in the high desirability condition (*M* = 4.41, SD = 1.63), indicating that the manipulation was successful.

**Figure 5:**
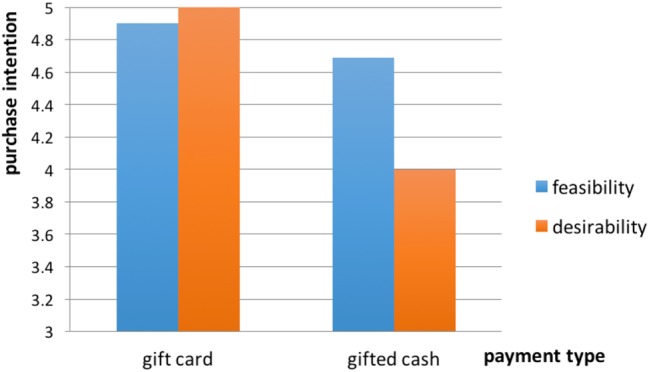
Interaction of product type and payment type.

For the four experimental conditions, we conducted a GLM analysis with type of payment (gift card vs. gifted cash) and product (feasibility vs. desirability) as the independent variables and purchase intention (*α* = 0.75) as the dependent variable. The main effect of mindset activated by payment type was significant [*F*(1, 147) = 5.221, *p* = 0.024]. Participants paying by gift card showed higher purchase intention (*M* = 4.95, SD = 1.57) for the remaining options than those paying with gifted cash (*M* = 4.33, SD = 1.76). Also consistent with our expectation (H4), the interaction between mindset activated by payment and initial product was significant [*F*(1, 147) = 2.26, *p* = 0.032]. Participants in the high desirability condition showed higher purchase intention when they paid by gift card (*M* = 5.00, SD = 1.47) relative to those paying with gifted cash [*M* = 4.00, SD = 1.75, *F*(1, 74) = 7.275, *p* = 0.008]. In contrast, the effect of payment was not significant for participants in the high feasibility condition [*F*(1, 73) = 0.51, *p* = 0.476] (see Figure 5).

Gift card recipients were more likely to adopt the which-to-buy mindset, thinking about when, where, and how to redeem the card, while those who received the gifted cash intended to put it into their own account and had to consider whether to spend it. Thus, consumers paying with gift cards show higher intention to buy the residual option compared with those using the gifted cash. The effect of payment type was moderated by product type. When the product is high in feasibility, participants construe the purchase task at a lower level. Even when they paid in gifted cash, the purchase intention did not decline sharply. When the product was high in desirability, participants tend to focus on high construal level information and consider whether to buy. Therefore, the purchase intention toward the remaining options decreased more sharply when participants were paying with gifted cash compared to gift cards.

## General Discussion

The present research proposes that the type of mindset can influence consumers’ construal level and thus the purchase intention toward the remaining options in a non-comparable choice set. Four studies provided converging support for this conclusion and the underlying hypothesis. The first study tested the main prediction that consumers with an activated which-to-buy mindset show higher purchase intention for remaining options relative to those with an activated whether-to-buy mindset. Supporting evidence for the construal level explanation arose through study 2; consumers with an activated which-to-buy mindset tend to construe at a lower level than those with an activated whether-to-buy mindset. The subsequent two studies examined two context factors of mindset: shopping cart state and payment. Study 3 demonstrated that initial purchasing easily activated the which-to-buy mindset and increased the sequential purchase intention of the remaining options. When the initial purchase was habitual, the effect was undermined as the habitual purchase could not prime the which-to-buy mindset. Study 4 found that gifted cash recipients thought more in a whether-to-buy mindset, thus decreasing their purchase intention for remaining options. If the product was shown to be high in feasibility, the effect was weaker as high feasibility products would involve a lower level of construal.

The results have several theoretical contributions. First, this is one of the first studies to focus on the unavailable effect in the non-comparable choice set, in which the decision principle is different from the comparable choice set (Parker and Schrift, 2011). If consumers cannot compare and rank the features of options in the set, then what affects their purchase decision for the remaining options? We identify the influence of the different types of mindset.

Second, Xu and Wyer (2007) found that a which-to-buy mindset would increase the likelihood of subsequent purchasing. We extend this research by applying it to the unavailable context choice and focus on the purchasing of remaining options where unavailable options exist.

Third, we extend the relationship between the theory of goal-related behavior (Srull and Wyer, 1989) and construal level (Liberman et al., 2007). We have shown that consumers with an activated which-to-buy mindset tend to process information at a lower construal level, while those with an activated whether-to-buy mindset construe at a higher level.

Further, we find two manipulation methods that activate different mindsets during the purchasing process. We find that shopping cart state represents different purchase stages and primes different type of mindsets. Consumers with empty shopping carts show lower purchase intention compared with those who have bought something. The initial purchase type was the boundary condition. When the initial purchase is habitual, the effect of mindset activated by shopping cart effect is weakened.

The other important priming method is the type of payment; paying by gifted cash (vs. gift card) decreases the purchase intention for the remaining options, since it activates the whether-to-buy mindset rather than the which-to-buy mindset. The boundary condition is the product type. When the product is shown to be high in feasibility, the effect of mindset activated by payment effect is undermined.

From a managerial perspective, these results offer implications for influencing retail shoppers faced with unavailable options in a common non-comparable choice set. First, when there are unavailable options in a non-comparable choice set, shopping guides are recommended to ask questions about preference, which can activate the which-to-buy mindset of consumers, thus increasing their intention to go for one. While in the online context, the unavailable information ought to be presented after consumers choose the option, so that they stay in a which-to-buy mindset. If the unavailable information is presented before consumers choose the non-comparable feature, they are still in the whether-to-buy mindset, and the incomplete structure of the choice set will increase their intention of forgoing.

Second, the recommendation of alternatives to unavailable products may be more readily accepted after consumers have put something in their shopping cart. Managers need to consider the exposure position of products and exhibit the unavailable choice sets on a further zone in the shopping route, so that consumers have more opportunities to put something into their cart. Managers should also consider the product type during the design of a recommendation system or route as this is a boundary condition.

Third, the payment type is important for managers to consider when developing their strategy. For example, when some options are not available during the later period of a sale, retailers could conduct promotions to encourage consumers using gift cards and thus increase their purchase intention for the residual products. The gift card promotion will be more efficient for those products with high desirability than those with high feasibility.

The present work has certain limitations. Future studies should be conducted in broader product categories to provide further support that the results were not due to the characteristics of the product category itself. Furthermore, our studies were all undertaken in a laboratory. Using real shopping area settings would increase the outside validity of the experiment results. Another fruitful area for future research is to identify other potential context variables that influence consumers’ mindsets while shopping.

## Ethics Statement

The experiments are conducted in several universities in Beijing. This study was approved by the Human Research Ethics Committee of Tsinghua University, and written informed consent was obtained from all subjects. As rewards, they were given gifts or credits. All the experimental data are only used for academic research and protected strictly.

## Author Contributions

JT and RC contributed to conceptualization. JT and RC contributed to data curation. JT, RC, and FH contributed to methodology. JT wrote the original draft. JT and FH wrote the review and edited.

### Conflict of Interest Statement

The authors declare that the research was conducted in the absence of any commercial or financial relationships that could be construed as a potential conflict of interest.
